# Sleep quality and creativity in Chinese college student during the COVID-19 pandemic: The mediating role of executive function

**DOI:** 10.3389/fpubh.2022.987372

**Published:** 2022-10-13

**Authors:** Botang Guo, Yue Song, Lu Zhao, Xinhui Cheng, Hanze Ma, Xiaohui Qiu, Xiuxian Yang, Zhengxue Qiao, Erying Zhao, Tianyi Bu, Jiarun Yang, Rupam Mishra, Yanjie Yang, Jiawei Zhou

**Affiliations:** ^1^Psychological Science and Health Management Center, Harbin Medical University, Harbin, China; ^2^Department of Student Affairs, Mental Health Education Center, Harbin Normal University, Harbin, China; ^3^Department of Human Resource Management, Health Management College, Harbin Medical University, Harbin, China; ^4^Academy of Educational Sciences, Heilongjiang University, Harbin, China

**Keywords:** COVID-19, creativity, sleep quality, executive function, mediation, college student

## Abstract

**Background:**

COVID-19 has impacted adolescents' interpersonal relationships, life attitudes, and mental health during the past 3 years. However, previous studies predominantly focused on negative problems, while few studies assessed the situation of teenagers from the perspective of positive psychology. Therefore, this study explores the creativity level of Chinese college students during the COVID-19 pandemic, the relationship between sleep quality and creativity, and the mediating role of executive function.

**Method:**

A cross-sectional study was conducted across six colleges in Heilongjiang in China, with a sample of 4,258 college students recruited *via* stratified cluster sampling. Data were collected through an online survey. A mediation model was constructed, and SPSS PROCESS macro was used to analyze the data.

**Results:**

The creativity score of Chinese college students during the COVID-19 pandemic was 106.48 ± 13.61. Correlation analysis demonstrated that sleep quality correlated negatively with creativity (*r* = −0.08, *P* < 0.01) but positively with executive function (*r*=0.45, *P* < 0.01), whilst executive function correlated negatively with creativity (*r* = −0.10, *P* < 0.01). Moreover, the mediation model revealed that executive function partially mediated the relationship between sleep quality and creativity in college students (indirect effec*t* = −0.017, *SE* = 0.004, 95% *CI* = [−0.025, −0.008]). Executive function accounted for 48.6% of the variance in college students' creativity.

**Conclusion:**

School administrators should implement measures such as sleep education to enhance students' sleep quality. Concurrently, curriculum and assessment implementation should enhance executive function. Such measures can contribute to improved student creativity, thus helping students overcome the negative emotional impact of the COVID-19 pandemic.

## Introduction

The COVID-19 outbreak disrupts people's lives and adversely impacts individuals physical and mental health (1). College students have attracted much attention as a group of interest during the pandemic. Long-term isolation from studying at home impedes college students' social and communication skills development, produces several negative psychological burdens (such as loneliness, anxiety, depression, fear, etc.), and hinders brain and personality development (2). Creativity is the ability to generate novel and useful schemes, ideas, or inventions, and it is of great significance to individual development and social progress (3). Humanism and positive psychology suggest that creativity is closely related to happiness and that creativity is an important means to psychological happiness and self-realization (4). Related research noted that individuals with high creativity during the COVID-19 outbreak cultivated more positive emotions, thus leading to lower perceived stress and better coping with the pandemic's negative effects (5). Hence, enhancing college students' creativity is an effective method to increase psychological adjustment and psychological coping ability (6), which is conducive to enhancing college students' positive emotions to deal with the negative emotions generated during the pandemic.

Studies have shown that sleep correlates with two different types of memory and that this association is fundamental to creativity and problem-solving (7). A survey showed longer bedtimes or wake times, more sleep disorders, and poorer sleep quality compared to the pre-pandemic baseline, albeit individuals' sleep duration increased (8, 9). Studies have shown that adolescents and young adults are the groups whose sleep was most severely affected by the pandemic (8). Distance learning and pandemic prevention and control measures increase college students' screen time while decreasing the frequency of outings and physical activity, which may disturb sleep quality and biological rhythm (10), thereby impeding replenishment of cognitive resources consumed during the day, inhibiting divergent thinking activities and negatively affecting creativity (11). Out of several literature studies, the majority focus on the abnormal psychological state of college students during the pandemic. However, few studies have explored the relationship between sleep status and creativity among college students during the pandemic. Hence this study employs positive psychology to explore whether sleep quality is significantly correlated with college students' creativity during the pandemic period, as well as the potential mechanism of this relationship, in order to provide practical recommendations for college students to cope with negative emotions during the pandemic period.

Sleep quality refers to the overall subjective satisfaction of individuals in sleep activities (12). It is a significant physical and mental resource (13). For students and workers, sleep contributes considerably to the recovery of psychological resources essential for self-regulation (14). According to studies on the ego depletion theory in the context of sleep (15, 16), after individuals complete their daily tasks, their ability to control their resources decreases; conversely, good sleep quality contributes to the restoration of control over resources depleted during daytime work, resulting in more efficient dedication of physical and mental resources to work (17). The creativity component theory posits that individuals express creativity in response to external environmental pressure (18), which can result in resource depletion. Depleted self-control resources may be detrimental to the generation of creative behaviors. High-quality sleep replenishes individuals' self-control resources, enhances their positive emotional experience and thinking fluency, and is conducive to active and creative problem-solving. Conversely, impaired sleep quality negatively impacts psychological, physiological, and brain functions, especially attention and divergent thinking (19). Therefore, we hypothesize that sleep quality is positively correlated with creativity.

Numerous scholars have verified the relationship between sleep quality and creativity, but the underlying mechanisms of this relationship require further exploration. Executive function is a complex cognitive process in which individuals implement targeted behaviors flexibly and coordinate the activities of multiple cognitive subsystems (20), including three major components, namely cognitive flexibility, working memory, and inhibitory control (21). COVID-19 has placed tremendous psychological pressure on people (22), leading to alterations in adolescents' emotions, behaviors, and other daily activities (23, 24). Studies have shown that chronic and acute stress affect individual cognitive processes controlled by the prefrontal cortex (PFC) (25), thereby impacting processes and functions within the executive system, including working memory, emotional self-regulation, cognitive flexibility, etc. (26). Sleep quality can have a major impact on the neural activity of the prefrontal cortex; hence sleep conditions may affect executive function.

Numerous cross-sectional studies have examined the relationship between sleep problems such as insufficient sleep duration and sleepiness in adolescents and academic performance, mental health, and cognitive functioning, and reported significant correlations between the two (27, 28).

In individuals who continuously engage in highly energy-consuming behaviors, timely replenishment of their resources is not achieved, causing a decline in executive functions; as a result, such individuals engage in shallow cognitive processing (29). Harrison and Home found that the executive function of the sleep-deprived group was significantly lower than that of the control group (30). Another study also confirmed that sleep deprivation affected executive function, wherein increasing sleep deprivation correlated with worsening task performance (31). A study examining the effects of working memory in adolescents following sleep deprivation demonstrated that sleep restriction for 5 consecutive days significantly decreased response speed in working memory tests. Concurrently, fMRI revealed brain function inhibition similar to that observed with complete sleep deprivation (32). Therefore, sleep quality may correlate positively with executive function.

Research shows that executive function is essential for generating creative thinking (33, 34). According to Norlander et al., the prefrontal cortex plays a central role in creativity (35). Thus, executive function and creative thinking share a common biological basis and are closely related (36). Benedek et al. demonstrated a positive correlation of creativity with cognitive control, whereby flexible adjustment of cognitive control contributes to creative performance (37). The literature also provides evidence that inhibition positively correlates with divergent thinking (38). Other studies have shown that individuals with high working memory ability are more likely to successfully overcome external interference and generate new ideas and strategies in creative thinking tasks (39). Vananina and Ansburg experimentally found that interfering information processing tasks (Hick task) were negatively related to creative potential, whereas for tasks requiring the suppression of interfering information (negative priming task and overall priority task), reaction times correlated positively with creativity (40, 41). Therefore, individual executive function enhancement can broaden creative thinking ability, enabling college students to improve their psychological coping ability in the face of pandemic changes, thereby ameliorating their physical and mental health. Therefore, we hypothesized that executive function is positively correlated with creativity and that executive function mediates the relationship between sleep quality and creativity.

### The present study

**Figure 1:**
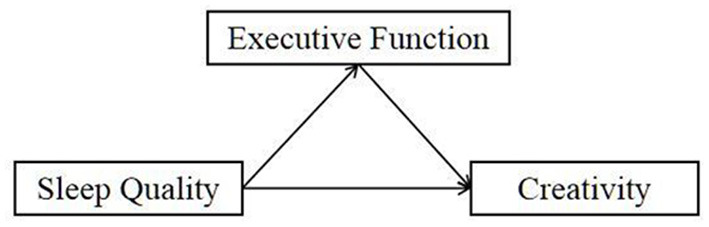
The hypothetical conceptual model.

Based on the literature review, this study proposed the following hypotheses (Figure 1):

Hypothesis 1. Sleep quality is negatively correlated with the creativity level of college students.
Hypothesis 2. Executive function is positively correlated with sleep quality and negatively correlated with creativity.
Hypothesis 3. Executive function mediates the relationship between sleep quality and creativity.

## Methods

### Participants and procedure

In December 2021, an online questionnaire survey was conducted among 4,500 college students from six universities in Heilongjiang Province, China, using a random cluster sampling method. Before the survey, consent was obtained from all participating college students. 4,258 valid questionnaires were collected (*M*_age_ = 19.88, *SD*_age_ = 1.87, Range_age_ = 17–26) for completion of the anonymous survey, with an effective rate of 94.62%. The survey included questions on demographic variables, sleep quality, executive function, and creativity levels. Among the total samples, 1,736 (40.8%) were male students, and 2,522 (59.2%) were female students. There were 2,367 (55.6%) single-child and 1,891 (44.4%) non-single-child family participants.

The online questionnaire used in this study contained uniform instructions to protect the informed consent of the subjects and ensure confidentiality of the content so as to improve the authenticity of the participants' responses. The survey system was set up prior to the survey. To preserve the accuracy of survey results, the questionnaire could only be filled in and submitted once from the same IP address to prevent multiple submissions by the same respondent. After the questionnaire was collected, the questionnaires with logical inconsistencies and evident irregularities were removed while valid questionnaires were retained.

### Measures

#### Sleep quality

The sleep quality of college students was measured using the self-rating sleep status scale (42). High total scores indicated poorer sleep quality. The scale consists of 10 items, divided into 5 grades. Participants rated each item on a five-point scale that ranged from one (never) to five (always). Chinese researchers recognize the self-rating sleep status scale as possessing good reliability and validity (43–45). In this study, Cronbach's α = 0.863, and the confirmatory factor analysis results (χ^2^/*df* = 90.511, CFI = 0.985, TLI = 0.924, RMSEA = 0.045) were within the acceptable range.

#### Executive function

The adolescent executive function scale was used as a measurement tool (46). The scale was composed of 21 items divided into 3 grades, with Cronbach's α = 0.972. The total score was the sum of each item's score. High total scores were indicative of subpar executive function; conversely, low total scores indicated better executive function. The reliability and validity of this scale have been authenticated by Chinese researchers (47, 48). Confirmatory factor analysis results (χ^2^/*df* = 46.859, CFI = 0.871, TLI = 0.857, RMSEA = 0.048) were within the acceptable range.

#### Creativity

The creativity of college students was measured with the Williams Creativity Tendency Scale (49). High total scores indicated a higher propensity for creativity. Total scores of more than 135 points, 120–134 points, 90–119 points, and less than 90 points represent excellent, good, average, and poor creativity, respectively. This scale consists of 50 items, each graded between 1 (completely inconsistent) to 3 (completely consistent), Cronbach's α = 0.972. The reliability and validity of this scale have been demonstrated in studies on college students in China (50, 51). Confirmatory factor analysis results (χ^2^/*df* = 129.73, CFI = 0.973, TLI = 0.940, RMSEA = 0.025) were within the acceptable range.

### Data analysis

All analyses were performed using SPSS 26.0 software. Analytic methods included descriptive statistics, regression analysis, correlation analysis, etc., and PROCESS macro analysis (Model 4) was employed to assess the mediating effect of executive function. The sample size in the model was set as 5,000, and the confidence interval (CI) was set as 95%. If the confidence interval did not contain zero, the effect was significant, and vice versa.

## Results

### Preliminary analysis

In this study, the average score generated by 4,258 students was (106.48 ± 13.61), which was representative of average creativity level. 107 (2.5%) participants had excellent creativity, 705 (16.6%) had average creativity, 3,217 (75.6%) had good creativity, and 229 (5.4%) had poor creativity. Descriptive analysis revealed that the creativity level of college students varies with age, single-child family status, registered residence, and subjective socioeconomic status (*P* < 0.05). See Table 1.

**Table 1:** Basic situation and creativity score of college students.

**Variables**	**Number of cases**	**Composition (%)**	**Creativity score**	***F/t***	***P***
Gender					
Male	1,736	40.77	106.94 ± 14.76	3.321	>0.05
Female	2,522	59.23	106.16 ± 12.76		
Age					
17–20	3,001	70.48	107.61 ± 13.60	2.414	< 0.01
21–23	991	23.27	104.45 ± 13.36		
24–26	266	6.25	101.28 ± 12.66		
The only child in a family					
Yes	2,367	55.59	107.19 ± 14.28	14.608	< 0.01
No	1,891	44.41	105.59 ± 12.68		
Registered residence					
Urban	2,453	57.61	107.57 ± 14.22	−6.129	< 0.05
Rural	1,805	42.39	104.99 ± 12.60		
Subjective socioeconomic status					
Low	581	13.64	104.06 ± 12.06	1.850	< 0.01
Middle	2,936	68.95	106.33 ± 13.48		
High	741	17.41	108.99 ± 14.84		

### Correlation analysis

The sleep quality score was (20.95 ± 6.27), the executive function score was (34.57 ± 9.18), and the creativity score was (106.48 ± 13.61). Correlation analysis showed that sleep quality was negatively correlated with creativity (*r* = −0.08, *P* < 0.01) but correlated positively with executive function (*r* = 0.45, *P* < 0.01), while executive function was negatively correlated with creativity (*r* = −0.10, *P* < 0.01). Specific results are shown in Table 2.

**Table 2:** Correlations among variables.

**Variables**	**(1)**	**(2)**	**(3)**	**(4)**	**(5)**
(1) Age	1				
(2) Sex	0.02	1			
(3) Sleep quality	−0.08^***^	0.03	1		
(4) Executive function	−0.10^***^	−0.03	0.45^***^	1	
(5) Creativity	−0.15^***^	−0.03	−0.08^***^	−0.10^***^	1

*** P < 0.01.

### Testing for mediation effect

**Figure 2:**
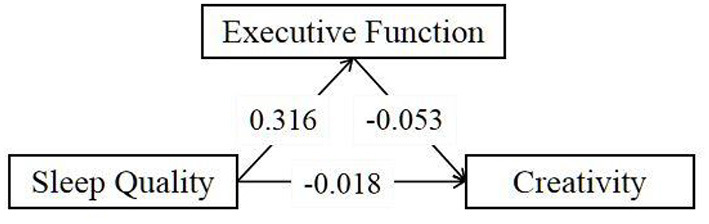
The confirmed mediation model.

Our results showed that sleep quality was negatively correlated with creativity (β = –0.035, *t* = –5.291, *P* < 0.001) and that the better the quality of sleep, the higher the level of creativity, supporting hypothesis 1. Process model 4 was used to test hypotheses 2&3, according to which executive function would mediate the relationship between sleep quality and creativity. As shown in Table 3, sleep quality was significantly positively correlated with executive function (β = 0.316, *t* = 33.228, *P* < 0.001) and significantly negatively correlated with creativity (β = –0.053, *t* = –4.952, *P* < 0.001), which implies that better executive function pairs with higher creativity level in college students. Executive function partially mediates the relationship between sleep quality and creativity (indirect effec*t* = −0.017, *SE* = 0.004, 95% CI= [−0.025, −0.008]), accounting for 48.6% of the total effect value (see Table 4). This supports the validity of hypotheses 2 and 3. The final confirmed mediation model was seen in Figure 2.

**Table 3:** Summary of hierarchical regression analyses predicting creativity.

	**Model 1**			**Model 2**			**Model 3**		
	**Y (Creativity)**			**M (Executive function)**			**Y (Creativity)**		
	**β**	***t***	***P***	**β**	***t***	***P***	**β**	***t***	***P***
X (Sleep quality)	−0.035^**^	−5.291	< 0.001	0.316^**^	33.228	< 0.001	−0.018^**^	−2.480	< 0.001
M (Executive function)	–	–	–	–	–	–	−0.053^**^	−4.952	< 0.001
*R^2^*	0.007			0.206			0.012		
	*F*_(1, 4258)_ = *P* < 0.001			*F*_(1, 4258)_ = *P* < 0.001			*F*_(2, 4257)_ = *P* < 0.001		

N = 4258;

** P < 0.01.

**Table 4:** Direct and indirect effects of sleep quality on creativity.

	**Effect size**	**Boot *SE***	**Boot CI lower limit**	**Boot CI upper limit**	**Relative effect size**
Total effect	−0.035	0.007	−0.048	−0.022	100%
Direct effect	−0.018	0.007	−0.033	−0.004	51.4%
Indirect effect	−0.017	0.004	−0.025	−0.008	48.6%

## Discussion

### Relevance of study results

The theory of two continuous models and the tenets of positive psychology posit that individuals also manifest positive changes when confronted with adversity (52, 53). Findings from this study aim to complement positive psychology reports on the physical and mental health of adolescents impacted by COVID-19. Firstly, we observed that the creativity level of Chinese adolescents during the pandemic was very low. Secondly, we found a significant correlation between sleep quality and creativity. Lastly, the executive function was identified as a mediator of this relationship.

The Chinese college students surveyed in this pandemic period study obtained medium creativity scores. Significant differences existed across individuals based on the comparison of creativity by grade, single-child status, household registration location, and subjective socioeconomic status. However, there was no significant difference across genders. As students progress in their studies, their development may be inhibited by practical considerations, resulting in lower creativity (54). Individuals from single-child households grow up with high-quality educational resources in an environment conducive to self-development. More opportunities to develop their creative potential are available to them than to individuals with siblings; hence, the former possess superior creative tendencies (55). In addition, there are significant geographical differences in the creativity levels of college students, whereby urban students are more creative than rural students. This indicates that the allocation of educational resources in China is uneven, while the environment strongly influences the development of creativity (56). There are also differences in the creativity of college students based on subjective socioeconomic status. In families with high socioeconomic status, parents are more educated, show more respect and understanding toward their children, often communicate with them, and allow them to express their own opinions, which facilitates the development of creativity (57). Conversely, families with low socioeconomic status experience more economic hardship, and family stress theory suggests that family economic stress increases parents' psychological stress, which leads to poor parenting behaviors such as low warmth and harsh punishment, which are not conducive to the development of creativity (58).

Sleep quality was found to be significantly negatively correlated with creativity. Poorer sleep quality in college students was associated with worsening creativity, consistent with previous research conclusions (59–61). According to the complementary learning system model, problem-related memories are transferred to the neocortex during sleep. When confronted with the problems again after sleep, neocortex activity is stronger while hippocampus activity is weakened, and memory reorganization is manifested in behavior, such that creative problem-solving ability is improved after sleep (62). According to the self-control depletion model, individuals will consume self-control resources when completing daily behavioral tasks, and lack of sleep hinders recovery of the consumed resources, thereby affecting the enthusiasm and creativity of individuals during daytime behavioral activities, resulting in a vicious cycle (13). High-quality sleep increases creativity by boosting individuals' willingness to invest resources and take risks when proposing new and useful ideas (17). The COVID-19 outbreak brought consequential disruptions to college students' daily life as remote studying from home became imperative (63). Among undergraduates with less constraint, there is a possibility of developing addictions to network gaming or entertainment programs which consume a large amount of physical and mental resources over time, thus affecting sleep patterns and quality (64), resulting in reduced daytime distribution of physical and mental resources. The inability to generate positive emotions to expand and regain self-control resources negatively impacts psychological (19), physiological and brain functions, especially attention and divergent thinking. The resulting reduction in the mental agility of college students during creative activities prevents them from reaching their full creative potential (11).

This study also examined the mediating role of executive function in sleep quality and creativity in college students. The results demonstrated a significant positive correlation between sleep quality and executive function as well as a significant negative correlation between executive function and creativity. Executive function partially mediated the relationship between sleep quality and creativity. A decline in sleep quality impedes replenishment of the individual's limited resources, thereby weakening inhibition ability and executive function in learning and life while reinforcing superficial cognitive processing (29). Studies have shown that short-term sleep deprivation can interfere with normal prefrontal cortex function (65, 66), while prefrontal cortex development is essential for the development of executive function (67, 68). Therefore, executive function is remarkably sensitive to alterations in sleep quality.

Although distance learning and pandemic prevention and control measures have provided increased opportunities for improving sleep quality, college students' prolonged video screen time and reduced outdoor activities may disrupt sleep quality and biorhythm (8), resulting in decreased cognitive flexibility during daytime activities, reduced ability to control thoughts and actions, and weakened creative thinking.

Creative thinking necessitates the contribution of executive function. These mental processes are related to the activity of the prefrontal cortex (36, 69). Neuroscience research also reveals involvement of the frontal lobe, posterior brain regions (70), and subcortical structures (71) in the creative process. Reduced executive function decreases thinking flexibility, weakens self-regulation and self-control, and limits the generation of novel ideas. Limitations in problem-solving capacity reduce creativity to a certain extent (72). During the pandemic, college students faced pressure from a disrupted pace of life with irregular work, rest hours, and sleep quality. This new irregularity impacts college students' remote learning, capacity for self-regulation and self-control, and thinking flexibility. Consequently, students become prone to negative emotions, with less incentive to partake in creative activities. Such students are also at risk of psychological disorders due to the strain on mental health during the pandemic. Creativity and executive function share a common physiological basis, and the development of creativity requires the strengthening of prefrontal cortex function. Impaired executive function can affect the performance of college students for tasks dependent on creativity (73). Working memory is an important component of executive function, and research indicates that sleep quality and working memory are interconnected (74). Research on rapid eye movement (REM) sleep and non-rapid eye movement (non-REM) sleep indicates that the REM sleep stage is principally responsible for memory reorganization by enhancing inter-memory connections and generating new connections, while the non-REM sleep stage contributes to rule extraction, formation of relational memory and memory integration, thereby complementing memory reorganization (75). During the pandemic, college students' sleep patterns were disrupted, and both REM and non-REM sleep were adversely affected to varying degrees; thus, executive function and creative problem-solving were impaired.

### Implications

This study expanded our understanding of the impact of sleep quality on college students' creativity in the face of public health emergencies by linking sleep quality to creativity. Unlike previous studies conducted in a controlled setting, data collection in this study occurred during the COVID-19 pandemic; hence, the results are more reflective of the real world. Furthermore, the present study analyzed the mediating role of executive functioning among college students. The results revealed that poor sleep quality affects executive function and negatively impacts students' creativity.

Applying measures based on the relationship between these three variables may enable teachers and school administrators to foster students' creativity and mental health more efficiently after the pandemic. Varying degrees of negative emotions in students seem inevitable after an pandemic stretching beyond 2 years. At such times, sleep education for college students should be enhanced, while curricula should pay attention to executive function training such that students can improve their adaptive skills, nurture their creativity and achieve more significant personal development.

### Limitations and future direction

Although all the hypotheses in this study have been verified, this study also contains limitations. Firstly, this study adopts a self-report method for the questionnaire survey. In the future, in addition to self-reports, other methods of data collection, such as evaluation of others, could be adopted to reduce deviation caused by a single research method. Secondly, this study adopted a cross-sectional research method which precludes the determination of a causal relationship between variables. Therefore, alternate methods such as longitudinal tracking should be applied in subsequent studies.

## Conclusion

In conclusion, this study revealed that Chinese college students' creativity level was average during the COVID-19 pandemic. This suggests that a joint contribution from educational institutions, families, and individuals may be warranted to improve the creativity level of college students during the pandemic. First and foremost, schools should be attentive to the sleep quality and mental health status of students compelled to study from home during the pandemic, adequately schedule time for learning and extracurricular activities, reinforce sleep education, ameliorate the psychological support system, and promptly intervene to treat students with serious psychological problems. Concurrently, educational institutions should train students' executive function *via* appropriate curricula and assessment measures. Furthermore, college students should adjust their mentality, sufficiently engage in outdoor sports, develop regular work and rest habits, and enhance their psychological coping ability by establishing and cultivating intimate relationships and maintaining emotional connections, with the aim of maintaining a state of good mental health.

## Data availability statement

The raw data supporting the conclusions of this article will be made available by the authors, without undue reservation.

## Ethics statement

The studies involving human participants were reviewed and approved by the Ethics Committee of Harbin Medical University. The patients/participants provided their written informed consent to participate in this study.

## Author contributions

BG, YS, and LZ wrote the main manuscript text. XC and HM prepared figures and tables. YY and JZ supervised the manuscript. XY, XQ, and ZQ investigated data. EZ and TB rewrote the manuscript. BG, JY, and RM revised the manuscript. All authors reviewed the manuscript. All authors contributed to the article and approved the submitted version.

## Funding

This research was funded by the National Natural Science Foundation of China (81903397) and the Heilongjiang Province Key Cultivation Think Tanks.

## Conflict of interest

The authors declare that the research was conducted in the absence of any commercial or financial relationships that could be construed as a potential conflict of interest.

## Publisher's note

All claims expressed in this article are solely those of the authors and do not necessarily represent those of their affiliated organizations, or those of the publisher, the editors and the reviewers. Any product that may be evaluated in this article, or claim that may be made by its manufacturer, is not guaranteed or endorsed by the publisher.
